# Egr-1 is a key regulator of the blood-brain barrier damage induced by meningitic *Escherichia coli*

**DOI:** 10.1186/s12964-024-01488-y

**Published:** 2024-01-17

**Authors:** Ruicheng Yang, Xinyi Wang, Hulin Liu, Jiaqi Chen, Chen Tan, Huanchun Chen, Xiangru Wang

**Affiliations:** 1 National Key Laboratory of Agricultural Microbiology, College of Veterinary Medicine, Huazhong Agricultural University, Wuhan, 430070 China; 2 Key Laboratory of Preventive Veterinary Medicine in Hubei Province, The Cooperative Innovation Center for Sustainable Pig Production, Wuhan, 430070 China; 3 Frontiers Science Center for Animal Breeding and Sustainable Production, Wuhan, 430070 China; 4 International Research Center for Animal Disease, Ministry of Science and Technology of the People′s Republic of China, Wuhan, 430070 China

**Keywords:** Meningitic *E. Coli*, Blood-brain barrier, Early growth response 1, Permeability, Neuroinflammation

## Abstract

Bacterial meningitis remains a leading cause of infection-related mortality worldwide. Although *Escherichia coli* (*E. coli*) is the most common etiology of neonatal meningitis, the underlying mechanisms governing bacterial blood-brain barrier (BBB) disruption during infection remain elusive. We observed that infection of human brain microvascular endothelial cells with meningitic *E. coli* triggers the activation of early growth response 1 (Egr-1), a host transcriptional activator. Through integrated chromatin immunoprecipitation sequencing and transcriptome analysis, we identified Egr-1 as a crucial regulator for maintaining BBB integrity. Mechanistically, Egr-1 induced cytoskeletal changes and downregulated tight junction protein expression by directly targeting VEGFA, PDGFB, and ANGPTL4, resulting in increased BBB permeability. Meanwhile, Egr-1 also served as a master regulator in the initiation of neuroinflammatory response during meningitic *E. coli* infection. Our findings support an Egr-1-dependent mechanism of BBB disruption by meningitic *E. coli*, highlighting a promising therapeutic target for bacterial meningitis.

**Supplementary Information:**

The online version contains supplementary material available at 10.1186/s12964-024-01488-y.

## Background

Bacterial meningitis remains a severe life-threatening central nervous system (CNS) infection [1]. *Escherichia coli* (*E. coli*), the most common Gram-negative bacillary organism, is the leading cause of neonatal meningitis [2]. Despite advances in antimicrobial treatment and supportive care, fatality rates range from 5 to 25%, with 25–50% of survivors suffering from neurologic sequelae [3]. To cause meningitis, bacterial pathogens must interact with and infiltrate the brain microvascular endothelial cells (BMEC) [4]. BMEC tightly regulate paracellular permeability via tight junction (TJ) proteins depending on the presence of several proteins including claudins, occludin, junctional adhesion molecules, and cell-selective adhesion molecules, which are linked to the actin cytoskeleton via cytoplasmic zona occludin family members (e.g., ZO-1, -2, and − 3) [5]. The preservation of blood-brain barrier (BBB) function relies on the integrity of TJ proteins and actin cytoskeleton. Meningitic *E. coli* induces the release of proinflammatory cytokines, chemokines, and certain toxic compounds, resulting in increased BBB permeability, pleocytosis, and serious CNS inflammatory injury [6, 7]. Additionally, evidence from experimental meningitis caused by other meningeal pathogens, such as Group B *Streptococcus* (GBS), *Streptococcus pneumoniae*, *Neisseria meningitidis* (*N. meningitidis*), *Haemophilus influenza* type B (Hib), and *Streptococcus suis* (*S. suis*), indicate that increased BBB permeability is a major contributor to CNS injury [8].

Early growth response 1 (Egr-1) is a Cys2-His2-type zinc finger transcription factor that is rapidly induced by cell-surface receptor signaling. It regulates gene expression in response to a variety of mitogenic signals in multiple cell types [9]. Egr-1 has a highly conserved DNA-binding domain with three zinc fingers that bind to the prototype target GC-rich consensus sequence 5′-GCGG(T)GGGCG-3′ [10]. Egr-1 may have multiple actions, including controlling synaptic plasticity, differentiation, wound repair, female reproductive capacity, inflammation, growth control, apoptosis, tumor progression, and vascular dysfunction [11]. Egr-1 is rapidly expressed in infectious diseases and participates in the pathogenic process. Egr-1 promotes the propagation of mouse hepatitis virus and herpes simplex virus 1 [12, 13], the reactivation of Epstein–Barr virus [14], human immunodeficiency virus type 1-mediated neurotoxicity [15], encephalitic viral-induced inflammation and cell death [16], and Venezuelan equine encephalitis virus-induced apoptosis [17]. In bacterial infections such as *Pseudomonas aeruginosa*, *Porphyromonas gingivalis*, and *Helicobacter pylori*, Egr-1 activation upregulates inflammatory mediators [18–20]. However, the potential molecular regulatory mechanisms of Egr-1 in bacterial meningitis remain unclear.

Here, we have demonstrated that infection of BMEC with meningitic *E. coli* induces the upregulation of the host transcription factor Egr-1, which acts as a facilitator for RhoA, Rac1, and Cdc42 activation and an inducer for vascular endothelial growth factor A (VEGFA), platelet derived growth factor subunit B (PDGFB), and angiopoietin like 4 (ANGPTL4) expression. This leads to breakdown of the BBB integrity through cytoskeletal changes and TJ proteins degradation. Our findings reveal a novel mechanism underlying BBB disruption by meningitic pathogens and elucidate how bacterial pathogens exploit host transcription factors to facilitate CNS infection.

## Methods

### Microbial strain and cell line

The *E. coli* strain PCN033 (GenBank: CP006632.1) was a highly virulent cerebrospinal fluid isolate first isolated in 2006 in Hunan Province, China. This strain was able to invade the host CNS, increase BBB permeability, and cause severe neuroinflammation (Fig. S1) [21]. human BMEC (hBMEC) were purchased from ScienCell (Carlsbad, CA, USA). Fetal bovine serum, 2 mM L-glutamine, 1 mM sodium pyruvate, MEM essential amino acids, MEM non-essential amino acids, vitamins, and 100 U/mL penicillin-streptomycin were added to the RPMI 1640 medium to supplement the hBMEC culture. Cells were cultured using dishes and flasks purchased from Jet Biofil (Guangzhou, China) [22]. The cells were incubated in a 5% CO_2_ humidified incubator at 37 °C.

### Bacterial infection of hBMEC

Confluent hBMEC were starved in serum-free media (1:1 Ham’s F-12 and medium 199 combination) for 16–18 h prior to infection. Overnight *E. coli* cultures were resuspended and diluted in serum-free medium before being added to the hBMEC at a multiplicity of infection of 10 to allow bacterial infection at 37 °C. Finally, TRIzol reagent (Invitrogen, Carlsbad, CA, USA) was used to extract RNA from hBMEC, and radio-immunoprecipitation assay (RIPA) lysis buffer (Beyotime, Shanghai, China) with protease inhibitor cocktail (MedChemexpress, Monmouth, NJ, USA) was used to extract protein from hBMEC.

### Transcription factor activation profiling analysis

A nuclear extract kit (Signosis, Santa Clara, CA, USA) was used to produce nuclear extracts in accordance with the instructions in the user guide. The cells were washed in phosphate buffer saline (PBS; pH 7.4) solution and lysed at 4 °C for 10 min in the extraction buffer I before being collected from the plates and centrifuged at 15,000 rpm for 3 min at 4 °C. After discarding the supernatant (cytoplasmic fraction), the pellet was resuspended in extraction buffer II and incubated for 2 h at 4 °C. The mixture was centrifuged at 15,000 rpm for 5 min at 4 °C, and the nuclear protein-containing supernatant was recovered and prepared for assays. The protein concentrations in brain lysates or cell lysates were determined using a BCA protein assay kit (Labgic Technology, Beijing, China). Every array test was carried out in accordance with the instructions provided in the TF activation profiling plate array II (Signosis, Santa Clara, CA, USA) user guide. The biotin-labeled probe mix was first treated with 10 ug of nuclear extract for 30 min at room temperature. Activated TFs were bound to the corresponding DNA binding probes. Following the separation of the protein/DNA complexes from unbound probes, the bound probes were eluted and hybridized with the plate that had been pre-coated with the capture oligos. The captured biotinlabeled probes were then detected with Streptavidin-HRP and measured using a microplate luminometer.

### Animal infection assay

The *Egr-1*^*−/−*^ mice were provided from the Jackson Laboratory (Bar Harbor, ME, USA). The specific pathogen-free C57BL/6 wild-type (WT) mice were obtained from Laboratory Animal Services Center at Huazhong Agricultural University. Mice aged 25 days were injected with *E. coli* strain PCN033 via the tail vein at 1 × 10^7^ colony-forming units (CFUs) diluted in PBS. At different times post-infection, the mice were anesthetized and the serum and brains were harvested for further assays. All efforts were made to ensure the ethical treatment of all experimental animals in this study and to minimize their suffering. Mice are euthanized when they exhibit severe neurological symptoms, such as convulsions, tremors, paralysis, and head tilt. To perform euthanasia, the mice undergo deep anesthesia and blood extraction.

### Real-time polymerase chain reaction (PCR)

Total RNA (500 ng) was subjected to reverse transcription with the HiScript II Q RT SuperMix (Vazyme, Nanjing, China). The generated mRNA was subsequently quantificated by using the HiPer SYBR Premix EsTaq (Mei5bio, Beijing, China) and the QuantStudio 3 RT-qPCR System (Applied BioSystems, Foster City, CA, USA). Primers used for real-time PCR are listed in Table S1.

### Western blot analysis

The equivalent protein samples were separated using 12% SDS-PAGE and electrophoretically transferred to polyvinylidene difluoride membranes. The blots were blocked for 2 h with Tris-buffered saline-Tween containing 2% bovine serum albumin before being incubated with primary antibodies against Egr-1 (Cell Signaling Technology, 4154, 1:1000, Danvers, MA, USA), Claudin-5 (Abcam, ab131259, 1:2000, Cambridge, MA, USA), vascular cell adhesion molecule 1 (VCAM-1) (Abcam, ab174279, 1:2000), ZO-1 (Proteintech, 21773-1-AP, 1:5000, Chicago, IL, USA), Occludin (Proteintech, 13409-1-AP, 1:2000), intercellular adhesion molecule 1 (ICAM-1) (Proteintech, 16174-1-AP, 1:1000), VEGFA (Proteintech, 19003-1-AP, 1:1000), ANGPTL4 (Proteintech, 18374-1-AP, 1:1000), β-actin (Proteintech, 66009-1-Ig, 1:20000), and PDGFB (GeneTex, GTX54575, 1:1000, Irvine, CA, USA). After that, the membranes were incubated with anti-mouse IgG (Biodragon, BF03008, 1:5000, Beijing, China) and anti-rabbit IgG (Biodragon, BF03001, 1:5000) with horseradish peroxidase conjugation. For the gray scale analysis, we first compared the target protein with its corresponding ß-actin and then compared it with the control sample (which was set to 1).

The activation of RhoA, Rac1, and Cdc42 were determined by using the RhoA/Rac1/Cdc42 Activation Assay Combo Biochem Kit (Cytoskeleton, BK030, Denver, CO, USA) following the instructions. Briefly, hBMEC were lysed in protease inhibitor-containing lysis buffer and incubated for 1 h at 4 °C with either 50 µg rhotekin-Rho-binding domain protein GST beads for RhoA detection or 10 µg p21-activated kinase 1 (PAK)-p21-binding domain protein GST beads for Rac1 and Cdc42 detection. After electrophoresis and membrane transfer, the samples were stained with anti-RhoA, anti-Rac1, and anti-Cdc42 antibodies, respectively [23].

### Chromatin immunoprecipitation sequencing (ChIP-seq) and integrated analysis

ChIP was performed on infected and control hBMEC with Egr-1 antibody by using SimpleChIP® Plus Enzymatic Chromatin IP Kit (Cell Signaling Technology, 9005) according to the manufacturer′s instructions. Briefly, cells in the dishes were fixed with formaldehyde to cross-link proteins and DNAs. Following micrococcal nuclease digestion, the cells were subjected to immunoprecipitation using Egr-1 (Cell Signaling Technology, 4154, 1:50) and IgG antibodies (negative control) respectively. The resulting products were treated with protease K and subjected to DNA isolation. Immunoprecipitated DNA was utilized for ChIP-PCR amplification with primers listed in Table S1.

ChIP-seq analysis was performed at Igenebook Biotechnology Co., Ltd. (Wuhan, China), following the protocol provided by the I NEXTFLEX® ChIP-Seq Library Prep Kit for Illumina® Sequencing (NOVA-5143-02, Bioo Scientific, Austin, TX, USA). Immunoprecipitated DNA was used for the construction of sequencing libraries, which were subsequently subjected to Illumina Xten sequencing using the PE 150 method [24]. MACS2 software (version 2.1.1.20160309) was used to call peaks by default parameters (bandwidth, 300 bp; model fold, 5, 50; q value, 0.05). If the summit of a peak is closest to the transcription start site of a particular gene, that peak will be assigned to that gene. The HOMER (version 3) software was used to predict the occurrence of motifs within peaks, with default settings for a maximum motif length of 12 base pairs. The raw ChIP-seq data generated in this study have been deposited in the BioProject database of the National Center for Biotechnology Information (NCBI; accession number PRJNA953929).

The enrichment analysis of Gene Ontology (GO) terms was performed by mapping the genes to NCBI GO annotations. This correspondence’s database was obtained from https://ftp.ncbi.nlm.nih.gov/gene/DATA/gene2, and Kyoto Encyclopedia of Genes and Genomes (KEGG) pathway annotation was performed using BLASTx against plant-specific sequences from the KEGG database. GO and KEGG enrichment analyses were performed using the hypergeometric test as implemented in the R phyper function.

### Clustered regularly interspaced short palindromic repeat (CRISPR)/CRISPR-associated protein 9 (Cas9) genomic editing

The CRISPR/Cas9 plasmid with a puromycin resistance gene was supplied by YSY Biotech (Nanjing, China). Human Egr-1 sgRNAs were synthesized and cloned into the CRISPR/Cas9 plasmid, and transfection was carried out as previously described [25]. The cells were transferred into 96-well plates using the limiting dilution method and incubated until a single-cell clone was formed. Western blot was used to confirm the identity of individual clones.

### Enzyme-linked immunosorbent assay (ELISA) and electrochemiluminescence assay

WT and *Egr-1*^*−/−*^ mice were intravenously challenged with a bacterial load of 1 × 10^7^ CFUs. After 9 h of infection, mouse brain extracts were harvested, and the levels of VEGFA, PDGFB, and ANGPTL4 levels in these extracts were quantified using mouse-specific VEGFA, PDGFB (Neobioscience, Shenzhen, China), and ANGPTL4 (Meimian, Yancheng, China) ELISA kits, following the manufacturers’ instructions. The levels of interleukin (IL)-1β, IL-6, tumor necrosis factor α (TNF-α), chemokine (C-C motif) ligand 2 (CCL-2), and chemokine (C-X-C motif) ligand 2 (CXCL-2) in brain extracts were measured using the Meso Scale Discovery kit (Meso Scale Diagnostics, Rockville, MD, USA), following the manufacturers’ instructions.

### Immunofluorescence analysis

The hBMEC samples were incubated with primary antibodies against ZO-1 (Proteintech, 21773-1-AP, 1:1000), Occludin (Proteintech, 27260-1-AP, 1:400), or Claudin-5 (Abclonal, A10207, 1:100) overnight at 4 °C. After thorough washing steps, the cells were subsequently incubated for additional hour with AF488-conjugated secondary antibody. The samples were counterstained with DAPI to visualize nucleus morphology. For cytoskeleton staining purposes, phalloidin was utilized to label F-actin of the cytoskeleton (MedChemexpress, Monmouth, NJ, USA).

Likewise, the mouse brain sections were incubated with primary antibodies against ICAM-1 (Proteintech, 16174-1-AP, 1:100), VCAM-1 (Abcam, ab134047, 1:2000), ZO-1 (Proteintech, 21773-1-AP, 1:1000), Occludin (Proteintech, 27260-1-AP, 1:400), or Claudin-5 (Abclonal, A10207, 1:100), and then with the Cy3-conjugated secondary antibody. The sections were then incubated with the primary CD31 (HuaAn, ER31219, 1:200, Hangzhou, China) antibody, followed by incubation with the secondary antibody fluorescein isothiocyanate before final nuclear staining with DAPI.

### BBB permeability assay

The BBB permeability was assessed by means of Evans blue dye (Sigma-Aldrich, St. Louis, MO, USA). C57BL/6 WT and *Egr-1*^*−/−*^ mice were challenged as described above, followed by intravenous injection of 500 µL Evans blue (5 mg/ml) and a circulation time of 10 min. After anesthesia, the mice underwent cardiac perfusion, and their brains were collected for photography to visualize the dye staining.

### Histopathological examination

The brain samples were collected, fixed in a 4% formaldehyde solution, and subsequently embedded in paraffin. Individual sections of 6-µm thickness were mounted on adhesive glass slides, dewaxed using xylene, and rehydrated through descending graded ethanol concentrations for hematoxylin and eosin staining.

### Statistical analysis

Data are expressed as mean ± standard deviation (mean ± SD), and the significance of differences between groups was determined by one-way analysis of variance or log-rank test (Mantel-Cox). A level of *p* < 0.05 (*) was considered significant, while *p* < 0.01 (**) or *p* < 0.001 (***) was considered extremely significant. GraphPad Prism Ver. 6.0 (GraphPad Software, La Jolla, CA, USA) was used for graph plotting and analysis.

## Results

### Induction of Egr-1 in brain endothelium during bacterial infection

**Fig. 1:**
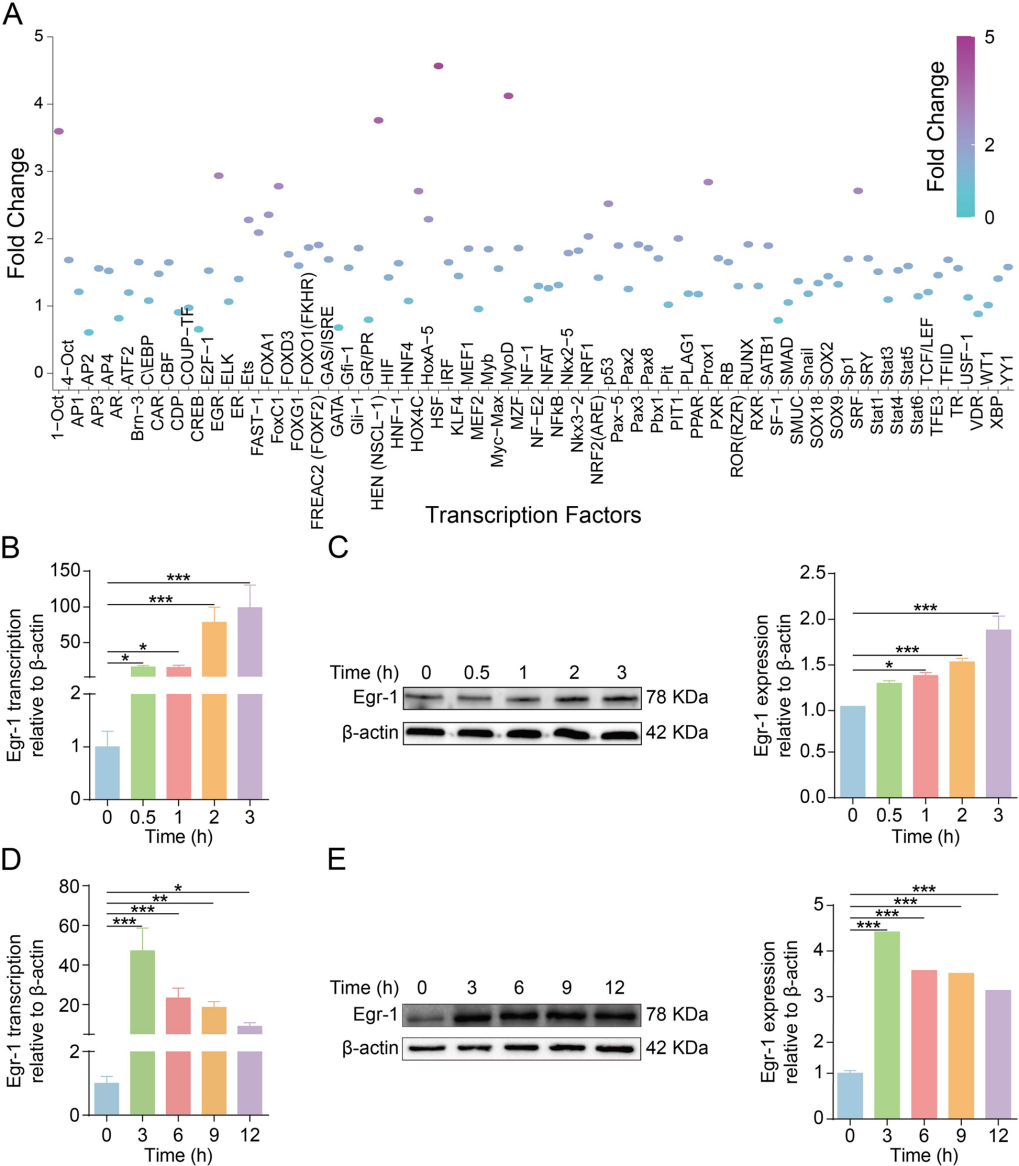
Meningitic *E. coli* infection massively increases Egr-1 activation in vitro and in vivo. **A** Plate array analysis of 96 TFs in hBMEC infected without or with meningitic *E. coli*. After 3 h of infection, the cells were subjected to nuclear extraction for the TF activation plate assay. Graph of gene array shows the activation or inhibition of 96 TFs in infected hBMEC compared with control hBMEC. **B** Real-time PCR determination of Egr-1 transcription in hBMEC upon infection. The transcription of β-actin was used as the internal reference. Data were presented as the mean ± SD from three independent experiments. *p < 0.05, ***p < 0.001. **C** Western blot analysis revealed increased expression of Egr-1 in hBMEC in response to infection. β-actin was used as the loading control, and densitometry was performed to analyze the differences. **D** Real-time PCR determination of Egr-1 transcription in microvessels isolated from the brains of bacterium-challenged mice at various time points. The transcription of β-actin was used as the internal reference. Data were presented as the mean ± SD from three independent experiments. *p < 0.05, **p < 0.01, and ***p < 0.001. **E** Microvessels isolated from the brains of bacterium-challenged mice at different time points were subjected to western blot analysis for expression of Egr-1. β-actin expression was used as the loading control, and densitometry of the bands was performed to compare differences

To investigate the transcription factor patterns induced by meningeal pathogens, hBMEC were treated for 3 h with or without meningitic *E. coli* infection. The most remarkable increases were observed in HSF, MYOD, HEN, OCT1, and Egr-1 activities (Fig. 1A). Notably, our previous mRNA sequencing results suggested that Egr-1 was the most significantly upregulated mRNA in meningitic *E. coli*-infected hBMEC (fold change, 36.61) [26], implying that Egr-1 plays an important regulatory role during meningitic *E. coli* interaction with the BBB. Further, we verified the activation of Egr-1 in vitro and in vivo. In the hBMEC model, we found that the mRNA transcription and protein level of Egr-1 showed a significant and time-dependent increase following the infection (Fig. 1B and C). By isolating the brain microvessels from the bacterium-challenged and control mice, we found that the transcriptional level of Egr-1 mRNA in mouse brain microvessels was significantly upregulated, with a greater than 40-fold increase at the early stage of infection (Fig. 1D). Meanwhile at the protein level, we observed a significant increase of Egr-1 in the brains of the infected mice that maintained a higher expression in the course of infection (Fig. 1E). These evidences indicate that meningitic *E. coli* challenge significantly activated Egr-1 in BMEC.

### The landscape of Egr-1-bound genes

**Fig. 2:**
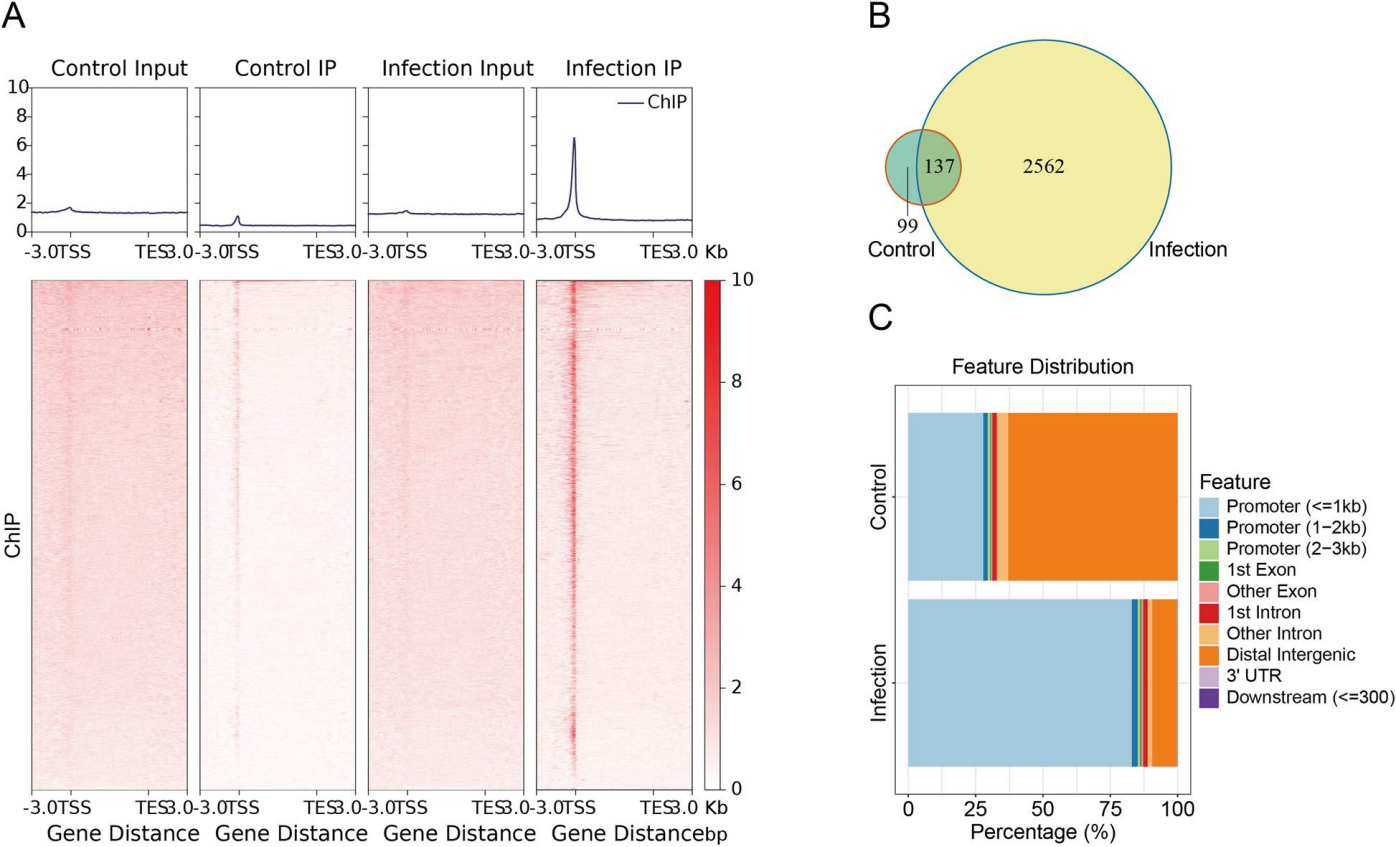
Identification of direct Egr-1 target genes and bioinformatics analysis. **A** Genome-wide occupancy of Egr-1 was investigated by ChIP-seq. ChIP-seq density heatmaps in bacterium-infected and control hBMEC for Egr-1 at TSS ± 3,000 bp. **B** Venn diagram showing the correlation analysis of Egr-1 peaks in infected and control hBMEC. **C** Distribution of genomic features of all accessible regions. Ten annotations were examined: promoter ( < = 1 kb), promoter (1 − 2 kb), promoter (2 − 3 kb), 1st exon, other exon, 1st intron, other intron, distal intergenic, 3′ untranslated region, downstream ( < = 300)

To define the role of Egr-1 in meningeal pathogen-induced BBB dysfunction, we performed ChIP-seq experiments targeting Egr-1 in meningitic *E. coli*-infected hBMEC. We observed a clear enrichment of Egr-1 binding at bacterium-induced open genomic sites, suggesting that these sites are regulated by Egr-1 (Fig. 2A). Statistical analysis revealed that the number of accessible peaks sharply increased in bacterium-infected hBMEC than in control. Between these two groups, 137 overlapping peaks were observed, with the infection and control groups displaying 2,562 and 99 unique peaks, respectively (Fig. 2B). To further characterize the genomic functional elements of accessible regions, hypergeometric optimization of motif enrichment (HOMER) was conducted to assess the genomic features [27]. More than 80% peaks in the infection group were predominately enriched in the promoter region, whereas most peaks in the control group were enriched in the distal intergenic region (Fig. 2C). These findings suggest that Egr-1 is the master transcription regulator in meningitic *E. coli*-induced BBB dysfunction.

### Association analysis of genes bound by Egr-1 and induced by infection

**Fig. 3:**
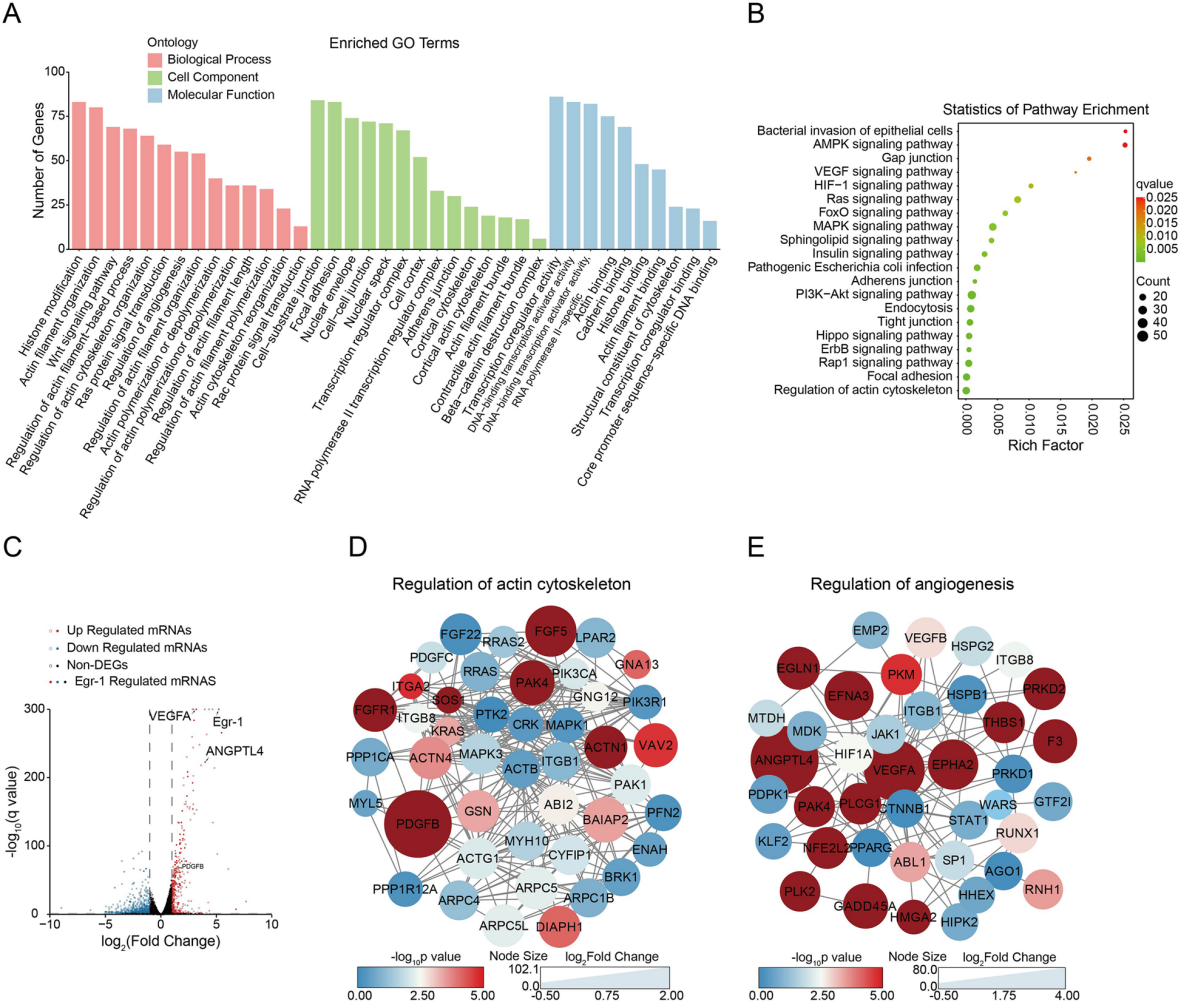
Bioinformatics analysis of Egr-1 target genes. **A** GO analysis of Egr-1 peaks in infected hBMEC. The x‑axis represents the name of the enrichment pathway. The y‑axis represents the targeted gene numbers corresponding to the GO terms. **B** Bubble diagram shows the KEGG pathways of Egr-1 peaks in infected hBMEC. The coloring of the *p* values indicates the significance of the rich factor. Circles indicate the target genes that are involved, with sizes proportional to the number of genes. The x‑axis represents the rich factor. The y‑axis represents the enrichment pathway name. **C** Volcano plot of the upregulated and downregulated mRNAs from hBMEC in the meningitic *E. coli* infection group compared with the control group. Red indicates upregulated mRNAs, blue downregulated mRNAs, and solid circles Egr-1 target genes. **D** Graphical illustration of the regulation of actin cytoskeleton-associated proteins using STRING (confidence score > 0.7). The coloring of the p values indicates the significance of the rich factor. Circle sizes indicate the log_2_Fold values. **E** Graphical illustration of the regulation of angiogenesis-associated proteins using STRING (confidence score > 0.7). The coloring of the p values indicates the significance of the rich factor. Circle sizes indicate the log_2_Fold values

To better understand the regulatory effects of Egr-1 in the development of bacterial meningitis, bioinformatics approaches were used to analyze the potential function of the unique peaks enriched in the promoter region in the infection group. A total of 2,322 genes were classified into “biological processes,” “cellular components,” and “molecular functions.” Within the biological processes class, these genes were mainly involved in histone modification, regulation of actin cytoskeleton organization, and regulation of angiogenesis. In the cellular components category, these genes were mainly divided into cell-substrate junction, focal adhesion, and cell-cell junction. Within the molecular functions category, these altered genes were primarily associated with DNA-binding transcription activator activity and transcription coregulatory activity (Fig. 3A). Moreover, the signaling pathways enriched by these 2,322 genes were determined with KEGG analysis. The results revealed several significantly enriched canonical signaling pathways, some known to regulate the meningitic *E. coli* invasion of the BBB (e.g., bacterial invasion of epithelial cells, pathogenic *E. coli* infection, endocytosis, PI3K-AKT signaling pathways, focal adhesion, and regulation of actin cytoskeleton), the permeability of the BBB (e.g., gap junction, adherens junction, TJ, and VEGF-, and HIF-signaling pathways), and neuroinflammation (e.g., MAPK- and ERBB-signaling pathways) (Fig. 3B). These results collectively indicate that meningitic *E. coli*-induced Egr-1 possesses a strong ability to damage BBB integrity and increase the inflammatory response during infection.

Our previous mRNA sequencing data revealed 366 genes for which the expression increased by ≥ 2-fold or decreased by ≤ 0.5-fold in hBMEC upon meningitic *E. coli* infection [26]. By combining these 366 significantly changed genes and 2,322 unique peaks enriched in the promoter region, we screened 111 candidate transcription targets that were directly regulated by Egr-1 (Table S2 and Fig. 3C). Since genes enriched in the regulation of the actin cytoskeleton and the regulation of angiogenesis pathways were closely related to the regulation of the BBB integrity, the gene sets in the regulation of the actin cytoskeleton and angiogenesis were extracted, and a protein–protein interaction network was generated with the STRING database. Among the clusters of interacting proteins involved in actin cytoskeleton regulation, PDGFB exhibited the most significant fold change (Fig. 3D), and among the clusters of interacting proteins involved in angiogenesis regulation, ANGPTL4 and VEGFA showed the most significant fold change (Fig. 3E). These findings suggest that PDGFB, ANGPTL4, and VEGFA are key targets for Egr-1 to affect BBB permeability.

### Meningitic *E. coli*-induced cytoskeleton alteration requires Egr-1 activation

**Fig. 4:**
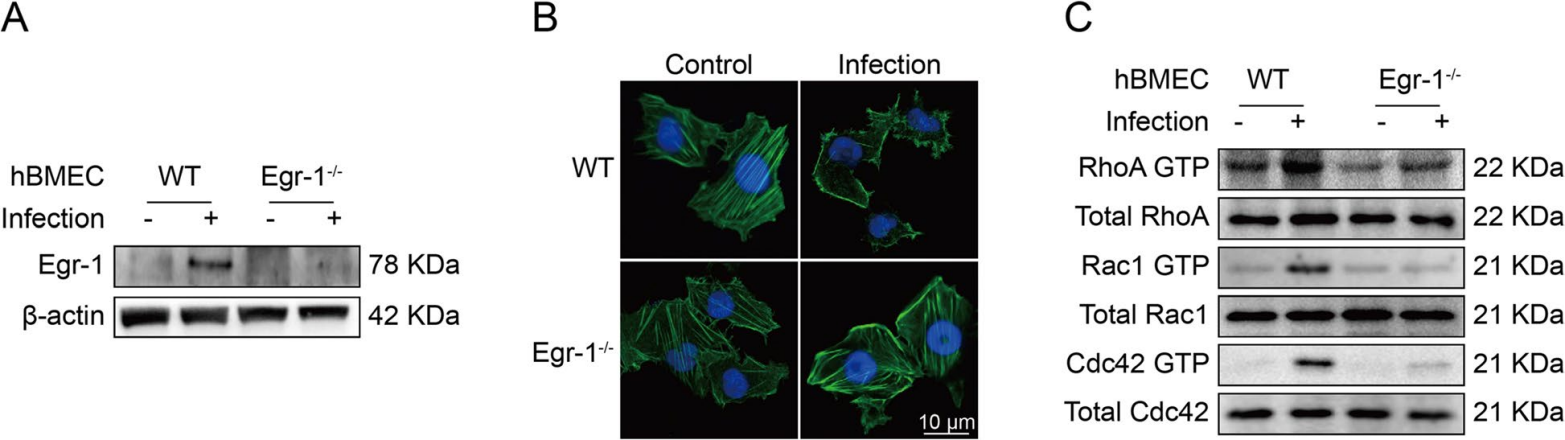
Egr-1 activation assists with meningitic *E. coli*-induced cytoskeleton fiber breakdown. **A** Western blot analysis of Egr-1 expression in WT and Egr-1^−/−^ hBMEC in response to infection. β-actin was used as the loading control. The induction of Egr-1 by meningitic *E. coli* was completely abolished in hBMEC, accompanied by Egr-1 knockout. **B** Cytoskeleton staining of WT and Egr-1^−/−^ hBMEC with or without bacterial infection. Phalloidin-labeled F-actin (green) and DAPI nuclear staining (blue). Scale bar indicates 10 µM. c Western blot analysis of RhoA, Rac1, and Cdc42 activation in WT and Egr-1^−/−^ hBMEC exposed to meningitic *E. coli*

The cytoskeleton rearrangement of BMEC is a key factor in driving BBB integrity impairment [28]. Therefore, we explored the possible relationship between Egr-1 activity and cytoskeleton alteration during infection. Egr-1 was genetically deleted in hBMEC using the CRISPR/Cas9 approach by introducing two guide RNAs. The infection-induced Egr-1 upregulation in endothelial cells was completely abolished, with no Egr-1 expression in the knockout cells (Fig. 4A). Moreover, we have observed that F-actin filaments in uninfected WT cells were well-assembled and internally stretched in a uniform and integral manner. In contrast, infected WT cells exhibited dispersed F-actin instead of filamentous structures, which were fragmentarily distributed throughout the cytoplasm. The normal cytoskeleton was disrupted and disintegrated, leading to cellular deformation. Contrarily, when Egr-1 was knocked out from the cells, no significant difference in the appearance of the cytoskeleton F-actin fibers was observed between cells with or without infection (Fig. 4B). Subsequently, we investigated the mechanism by which activated Egr-1 promotes cytoskeletal rearrangement in BMEC. In this regard, canonical Rho-GTPases, including RhoA, Rac1, and Cdc42, are key regulators of cell cytoskeleton [29]. Notably, an increase in GTP-bound (active) forms of RhoA, Rac1 and Cdc42 was observed in WT cells after 1 h of infection; however, such activation was largely prevented in Egr-1-knockout cells (Fig. 4C). These findings suggest that meningitic *E. coli*-induced cytoskeletal alterations in hBMEC require Egr-1 expression.

### VEGFA, PDGFB, and ANGPTL4 expression are regulated by Egr-1

**Fig. 5:**
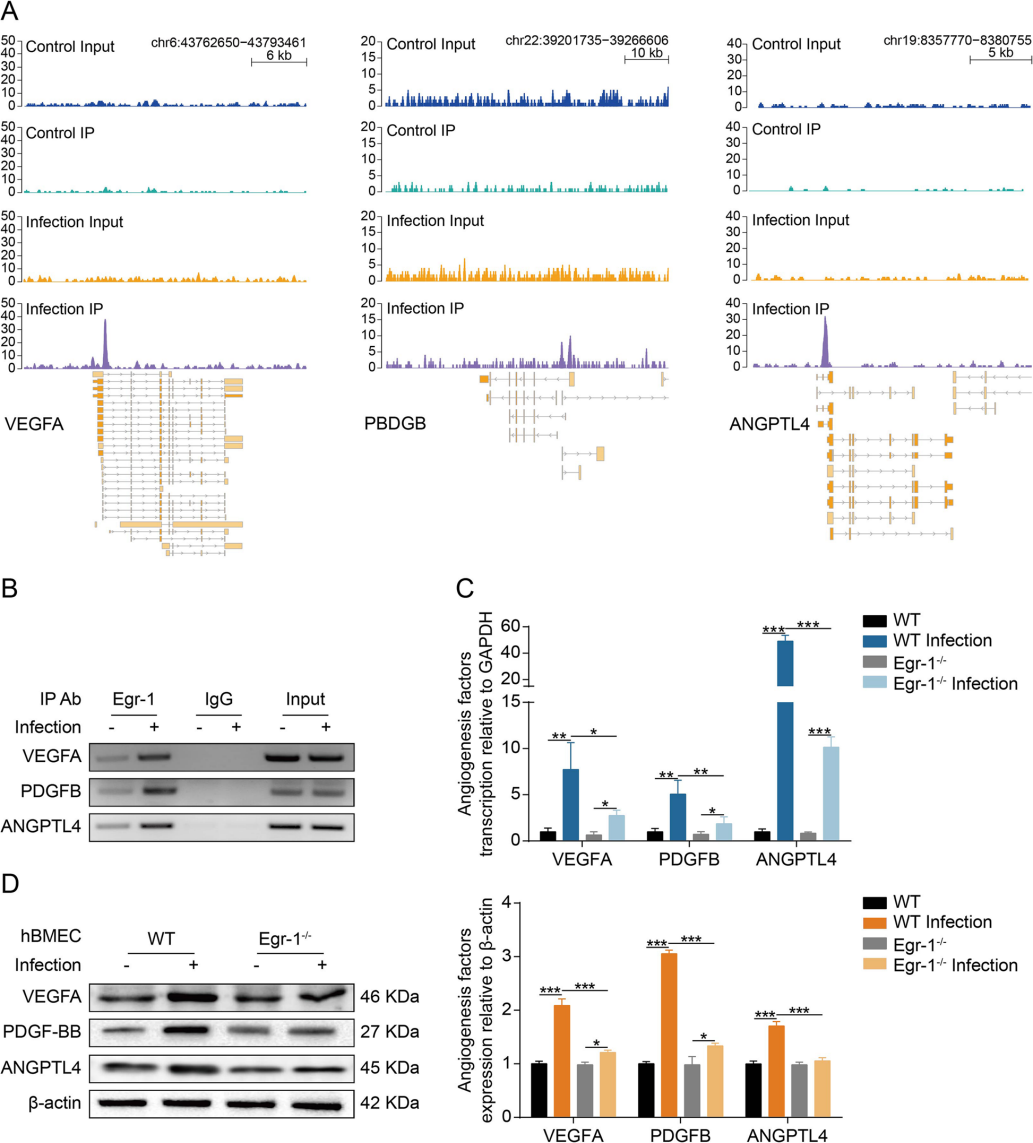
Egr-1 is required for VEGFA, PDGFB, and ANGPTL4 induction. **A** ChIP-seq binding peaks on VEGFA, PDGFB, and ANGPTL4 gene promoter for Egr-1. **B** ChIP-PCR validation of the Egr-1 binding to the VEGFA, PDGFB, and ANGPTL4 promotor in hBMEC infected by meningitic *E. coli*. The nuclear extracts were immunoprecipitated with anti-Egr-1 antibody or normal IgG. Input refers to the same dose of nuclear extract administered prior to immunoprecipitation. **C** Real-time PCR analysis of VEGFA, PDGFB, and ANGPTL4 transcription in WT and Egr-1-knockout hBMEC upon infection. The transcription of β-actin was used as the internal reference. Data were presented as the mean ± SD from three independent experiments. *p < 0.05, **p < 0.01, and ***p < 0.001. **D** Western blot analysis of VEGFA, PDGFB, and ANGPTL4 expression in WT and Egr-1-knockout hBMEC upon infection. β-actin was used as the loading control, and densitometry was performed to analyze the differences

Bioinformatics analysis suggests that Egr-1 regulates the actin cytoskeleton and angiogenesis via its potential target genes VEGFA, PDGFB, and ANGPTL4. The ChIP-seq data revealed the significant Egr-1-binding peaks in the promoter region of VEGFA (chromosome 6 position 43,771,366 − 43,771,763), PDGFB (chromosome 22 position 39,243,987 − 39,244,220), and ANGPTL4 (chromosome 19 position 8,363,766-8,364,203) in the infection group. In contrast, almost no binding peak was observed in the control group (Fig. 5A). Therefore, we hypothesized that VEGFA, PDGFB, and ANGPTL4 may be the potential target genes of Egr-1 in meningitic *E. coli* infection of hBMEC. Subsequently, we conducted a ChIP-PCR assay to confirm whether Egr-1 regulates VEGFA, PDGFB, and ANGPTL4 expression via direct binding to their promoters. Protein-DNA complexes were immunoprecipitated with antibodies against Egr-1 or normal IgG. An aliquot of non-immunoprecipitated chromatin was employed as the input control to show specificity. The ChIP-PCR results demonstrated the significant increase in Egr-1 binding to the VEGFA, PDGFB, and ANGPTL4 promoters after treating hBMEC with bacteria (Fig. 5B). Additionally, real-time PCR and western blot analysis showed that Egr-1 knockout partially suppressed VEGFA, PDGFB, and ANGPTL4 at the transcription and expression levels in infected hBMEC (Fig. 5C and D). These together demonstrate that Egr-1 activation is required for the upregulation of VEGFA, PDGFB, and ANGPTL4 in meningitic *E. coli*-infected hBMEC.

### Egr-1 is a crucial prerequisite for the disruption of TJ proteins

**Fig. 6:**
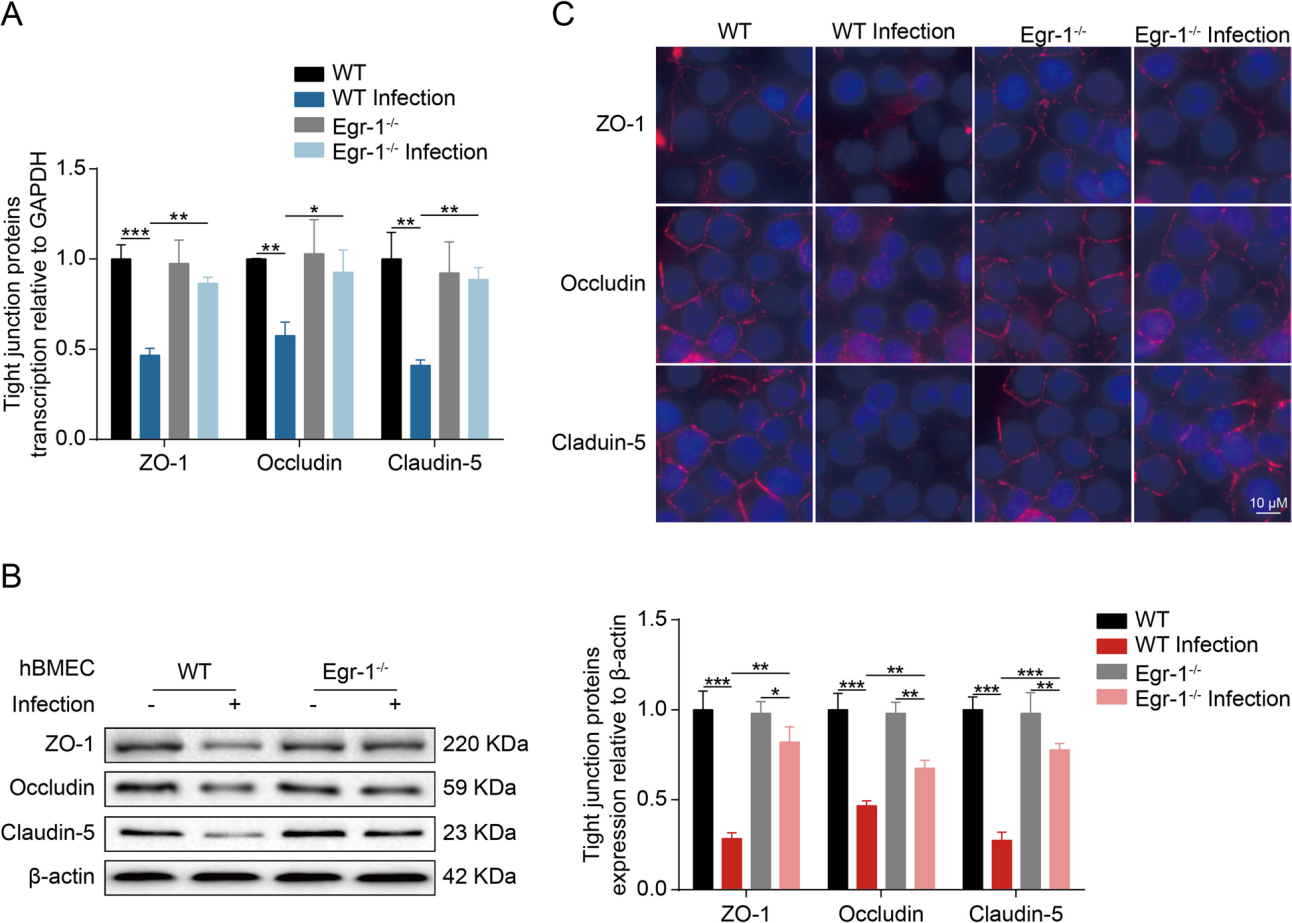
Egr-1 knockout in hBMEC suppresses meningitic *E. coli*-induced TJ protein degradation. **A** Real-time PCR analysis of ZO-1, Occludin, and Claudin-5 transcription in WT and Egr-1-knockout hBMEC upon infection. The transcription of β-actin was used as the internal reference. Data were presented as the mean ± SD from three independent experiments. *p < 0.05, **p < 0.01, and ***p < 0.001. **B** Western blot analysis of ZO-1, Occludin, and Claudin-5 expression in WT and Egr-1-knockout hBMEC upon infection. β-actin was used as the loading control, and densitometry was performed to analyze the differences. **C** Immunofluorescence analysis determining the distribution and expression of ZO-1, Occludin, and Claudin-5 in WT and Egr-1-knockout hBMEC upon infection. Nuclei were stained in blue with DAPI, while ZO-1, Occludin, and Claudin-5 were stained in red. Scale bar, 10 μm

Meningitic *E. coli* infection can induce VEGFA, PDGFB, and ANGPTL4 expression, aggravating infection-dependent BBB dysfunction [21, 30, 31]. In this study, we found that the induction of VEGFA, PDGFB, and ANGPTL4 by meningitic *E. coli* was largely prevented after Egr-1 knockout in hBMEC. Subsequently, we evaluated whether Egr-1 activation might affect the integrity of the hBMEC monolayer. We compared the regulation of Egr-1 on the TJ proteins, including ZO-1, Occludin, and Claudin-5 in both Egr-1^−/−^ and WT hBMEC in response to the infection. Although the downregulation of TJ proteins upon infection was not completely abolished, a significantly attenuated decrease in TJ proteins at both mRNA and protein levels in Egr-1-knockout hBMEC was observed compared with those in control hBMEC (Fig. 6A and B). Moreover, immunofluorescence analysis was performed to examine the distribution and expression of ZO-1, Occludin, and Claudin-5 in Egr-1^−/−^ and WT hBMEC upon infection. As shown, the TJ proteins were well-organized and distributed around the uninfected Egr-1^−/−^ and WT cells. However, these proteins became inconsecutive, irregularly distributed, or scattered around the WT cells upon bacterial infection, whereas this damage was almost completely prevented in the Egr-1^−/−^ hBMEC (Fig. 6C). Collectively, these findings demonstrate that the disruption of BBB integrity induced by meningitic *E. coli* infection is dependent on the activation of Egr-1.

### Egr-1 enhances BBB permeability by increasing VEGFA, PDGFB, and ANGPTL4 expression

**Fig. 7:**
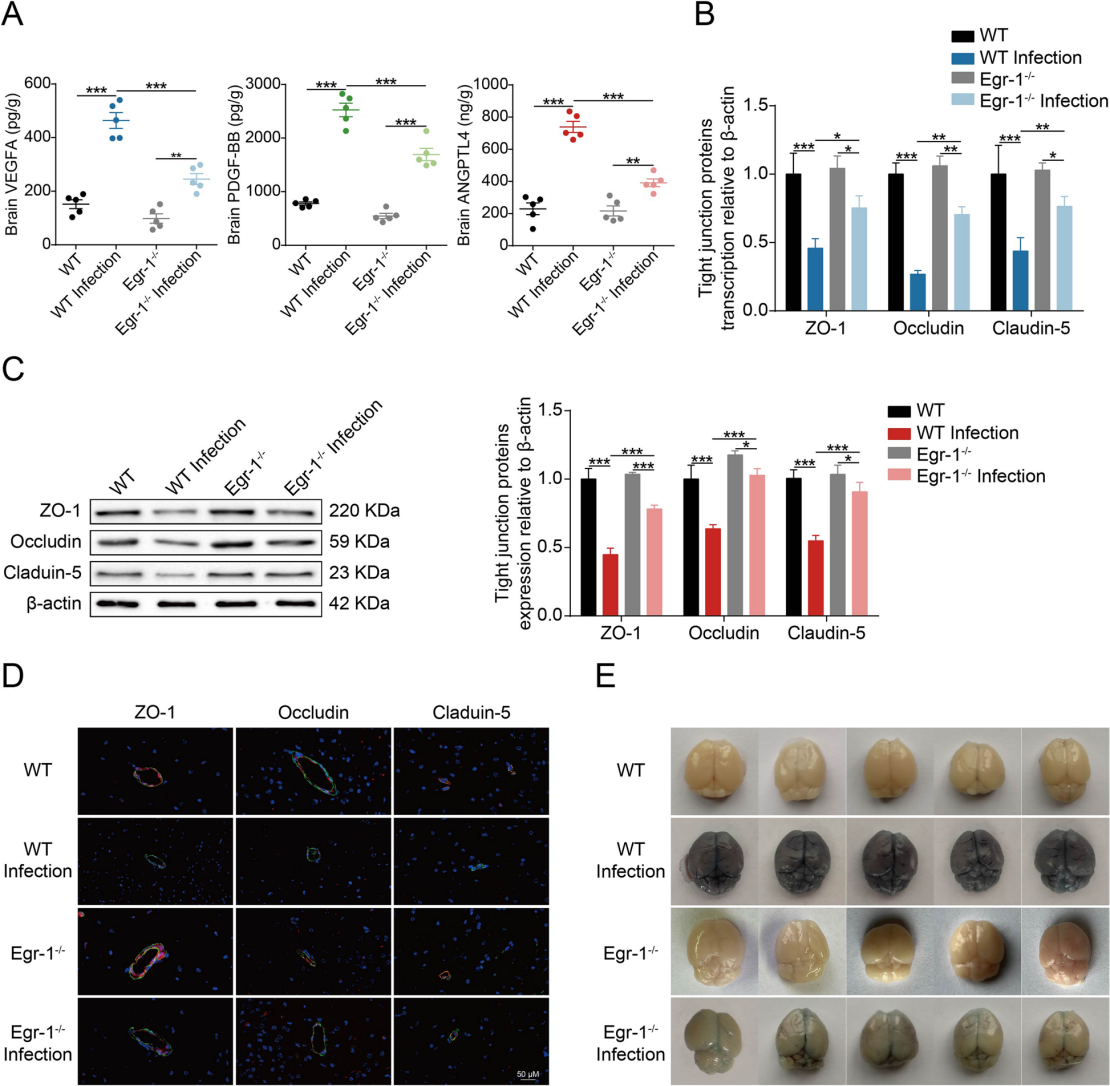
Egr-1 knockout in mice suppresses meningitic *E. coli*-induced BBB disruption. **A** ELISA analysis of VEGFA, PDGFB, and ANGPTL4 in brain lysates from challenged WT and *Egr-1*^*−/−*^ mice. Data were collected and presented as mean ± SD. *p < 0.05, **p < 0.01, and ***p < 0.001. **B** Real-time PCR analysis of ZO-1, Occludin, and Claudin-5 transcription in brains from challenged WT and *Egr-1*^*−/−*^ mice. The transcription of β-actin was used as the internal reference. Data were presented as the mean ± SD from three independent experiments. *p < 0.05, **p < 0.01, and ***p < 0.001. **C** Western blot analysis of ZO-1, Occludin, and Claudin-5 expression in brain lysates from challenged WT and *Egr-1*^*−/−*^ mice. β-actin was used as the loading control, and densitometry was performed to analyze the differences. **D** Immunofluorescence analysis of vascular endothelium integrity in infected WT and *Egr-1*^*−/−*^ mice. ZO-1, Occludin, and Claudin-5 were stained in red. CD31 was specifically applied for labeling the microvessels in green. The cell nucleus was stained in blue with DAPI. Scale bar indicates 50 μm. **E** Evans blue assay was used to evaluate BBB permeability in WT and *Egr-1*^*−/−*^ mice with or without meningitic *E. coli* infection (*n* = 5)

The *Egr-1*^*−/−*^ mice were used to evaluate the contribution of Egr-1 to VEGFA, PDGFB, and ANGPTL4 production in response to the infection. By using ELISA, we found that the infection-caused upregulation of VEGFA, PDGFB, and ANGPTL4 in the brains of *Egr-1*^*−/−*^ mice significantly decreased compared with that in WT mice (Fig. 7A). To further confirm that the TJ protein degradation in mouse brain vascular endothelial cells observed with meningitic *E. coli* infection was controlled by Egr-1, real-time PCR and western blot analysis were performed to detect the expression of TJ proteins in mouse brains. The transcription and expression levels of ZO-1, Occludin, and Claudin-5 in bacterium-challenged WT mice significantly decreased, whereas this degradation was significantly reversed in *Egr-1*^*−/−*^ mice (Fig. 7B and C). Meanwhile, immunofluorescence was performed to examine the distribution of ZO-1, Occludin, and Claudin-5 in infected WT mice and *Egr-1*^*−/−*^ mice. These TJ proteins were well-organized and distributed around the blood vessels in the uninfected WT mice and *Egr-1*^*−/−*^ mice. Results also indicated that the vascular endothelial layer became inconsecutively distributed, irregular or gapped in bacterium-infected WT mice, whereas these adverse effects of TJ proteins were well restored in bacterium-infected *Egr-1*^*−/−*^ mice (Fig. 7D). Additionally, Evans blue infiltration was used to assess the permeability of the mouse BBB. Evans blue entry into the brains of infected *Egr-*1^*−/−*^ mice was significantly lower than that of infected WT mice (Fig. 7E). Consequently, these data largely supported Egr-1 as an important contributor to BBB breakdown in *E. coli* meningitis.

### Contribution of Egr-1 to meningitic *E. coli*-induced neuroinflammation

**Fig. 8:**
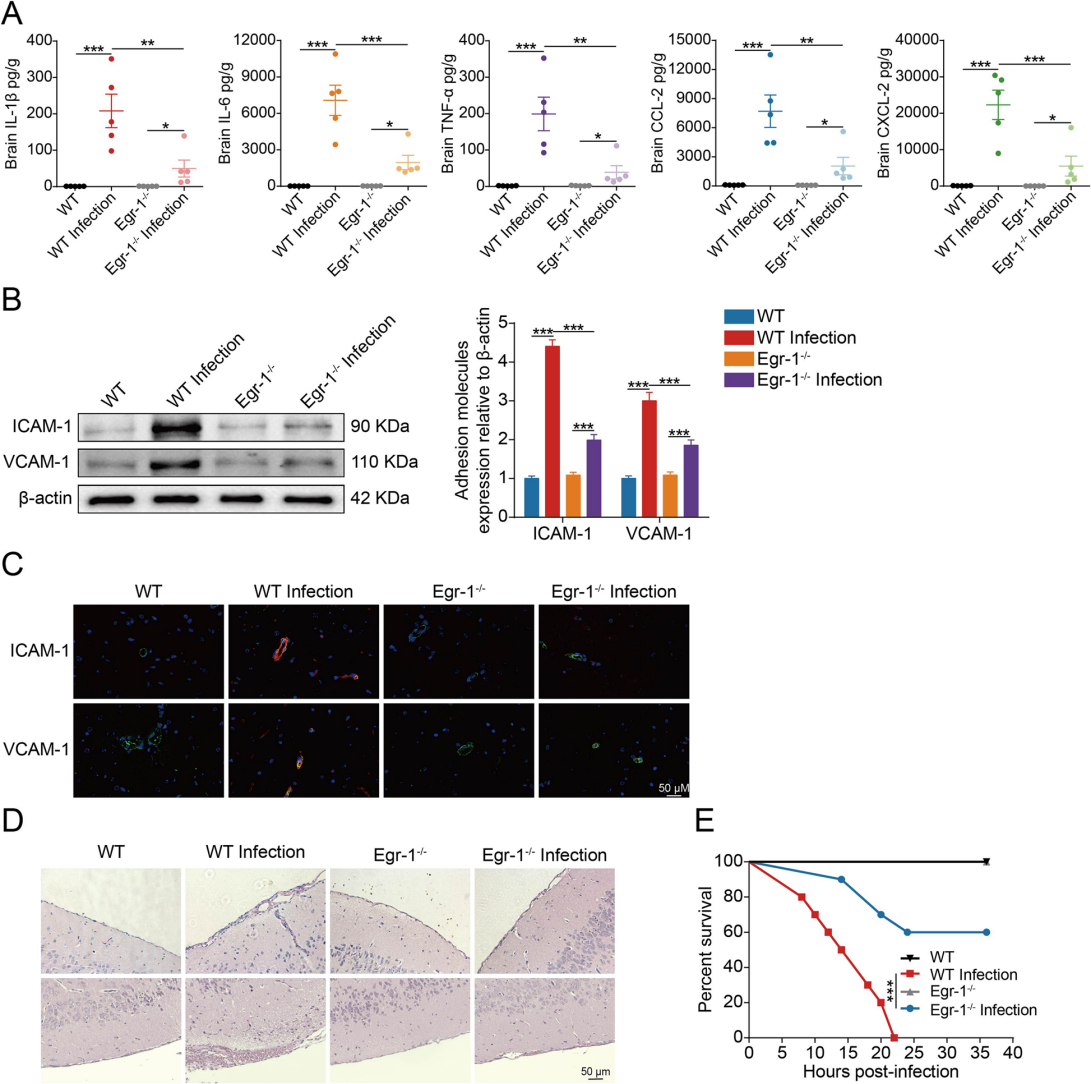
Egr-1 knockout in mice reduces neuroinflammation and improves the survival rate in meningitic *E. coli* infection. **A** Electrochemiluminescence analysis of IL-1β, IL-6, TNF-α, MCP1, and MIP2 in brain lysates from challenged WT and *Egr-1*^*−/−*^ mice. Data were collected and presented as mean ± SD. *p < 0.05, **p < 0.01, and ***p < 0.001. **B** Western blot analysis of ICAM-1 and VCAM-1 expression in brain lysates from challenged WT and *Egr-1*^*−/−*^ mice. β-actin was used as the loading control, and densitometry was performed to analyze the differences. **C** Immunofluorescence analysis of ICAM-1 and VCAM-1 in the brains of infected WT and *Egr-1*^*−/−*^ mice. Perivascular ICAM-1 and VCAM-1 were stained red. CD31 was specifically applied for labeling the microvessels in green. The cell nucleus was stained in blue with DAPI. Scale bar indicates 50 μm. **D** Histopathological examination of brain sections. The scale bar indicates 50 μm. **E** Survival of mice in each group was monitored for 36 h after tail vein injection of meningitic *E. coli*. Data was collected and shown as Kaplan–Meier survival curves (*n* = 10). Statistical analysis was carried out by log-rank (Mantel–Cox) test. ***p < 0.001

To further determine the effects of Egr-1 on CNS inflammation induced by meningitic *E. coli*, we subsequently assessed its ability to regulate cytokine and chemokine production in response to in vivo infection using electrochemiluminescence assay. We found that IL-1β, IL-6, TNF-α, CCL-2, and CXCL-2 levels in the brain were significantly augmented in bacterium-infected WT mice; however, the upregulation of these cytokines and chemokines was significantly reduced in bacterium-infected *Egr-1*^*−/−*^ mice (Fig. 8A). Leukocyte adhesion and trafficking across the BBB are dependent on the activation and expression of adhesion molecules such as ICAM-1 and VCAM-1 [30]. Therefore, ICAM-1 and VCAM-1 expression was analyzed in brain tissues from bacterium-infected WT and *Egr-1*^*−/−*^ mice. We observed an upregulation of ICAM-1 and VCAM-1 in brains derived from the bacterium-infected WT mice but not in the bacterium-infected *Egr-1*^*−/−*^ mice (Fig. 8B). We also analyzed ICAM-1 and VCAM-1 expression by immunofluorescence. The expression of ICAM-1 and VCAM-1 was elevated and co-localized with blood vessels labeled with CD34 in the bacterium-challenged WT mice, compared with the bacterium-challenged *Egr-1*^*−/−*^ mice (Fig. 8C). These evidences indicate that ICAM-1 and VCAM-1 upregulation induced by meningitic *E. coli* infection depends on the activation of Egr-1. Moreover, brain sections from control and meningitic *E. coli*-challenged WT and *Egr-1*^*−/−*^ mice were examined. Histopathological examination showed noticeable meningeal thickening and corresponding inflammatory cell accumulation after bacterial infection in WT mice, but this histologic lesion was ameliorated in *Egr-1*^*−/−*^ mice (Fig. 8D). Considering the detrimental impact of Egr-1 on BBB integrity and neuroinflammation exacerbation in mice infected with meningitic *E. coli*, we proceeded to investigate whether Egr-1 knockout could confer protection against meningitic *E. coli*-induced mortality in mice. As demonstrated, all mice in the uninfected WT and *Egr-1*^*−/−*^ group survived throughout the observation period. Conversely, a 100% mortality rate was observed in WT mice with bacterial infection. However, Egr-1 knockout provided protection from mortality in 60% of the *Egr-1*^*−/−*^ mice with bacterial infection (Fig. 8E). Collectively, these findings strongly support the involvement of Egr-1 in meningitic *E. coli*-induced neuroinflammation as well as mortality.

## Discussion

**Fig. 9:**
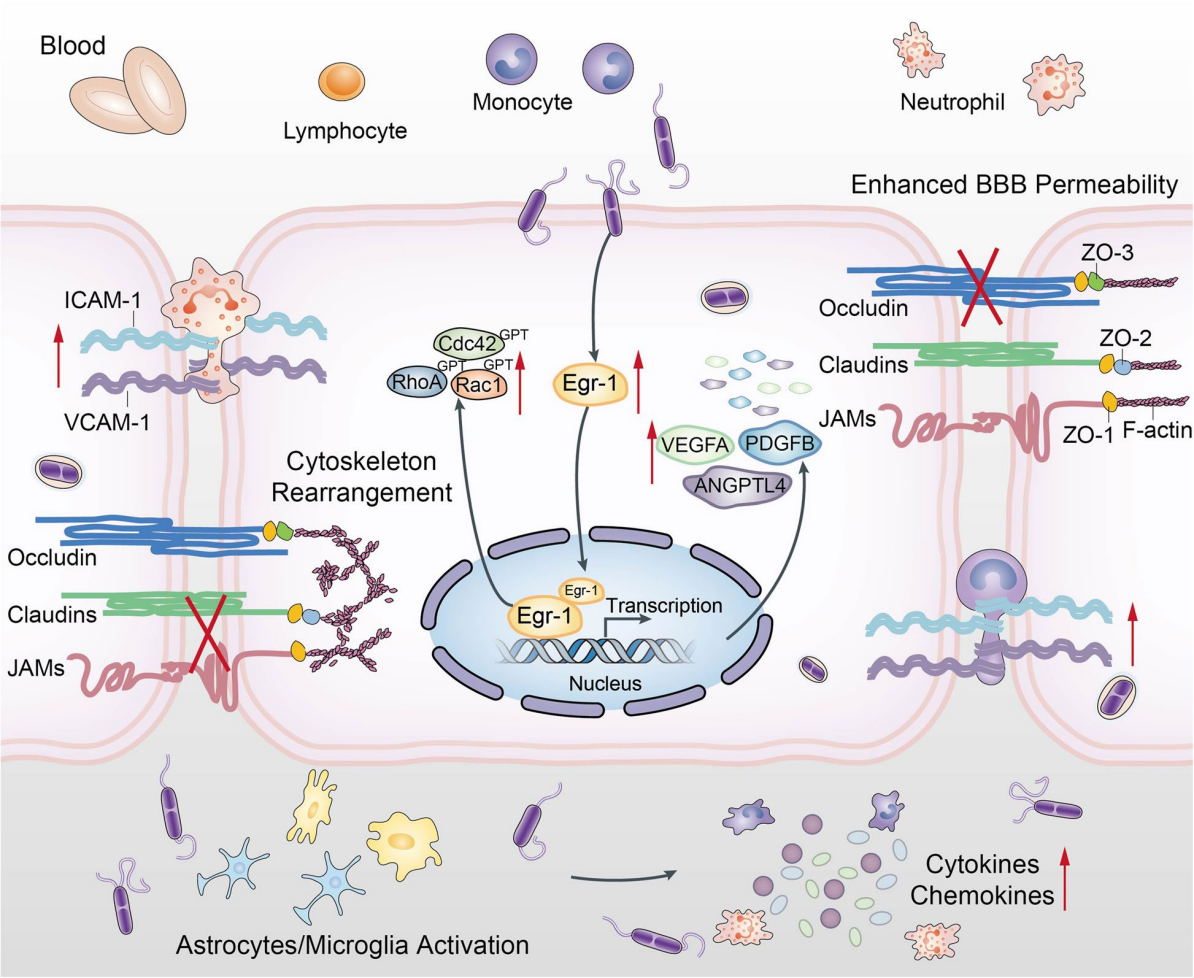
Schematic presentation of meningitic *E. coli* induction of Egr-1 for BBB disruption as well as neuroinflammatory responses. Infection of BMEC with meningitic *E. coli* rapidly induces expression of the host transcription factor Egr-1. Egr-1 facilitates the activation of RhoA, Rac1, and Cdc42 and promotes the expression of VEGFA, PDGFB, and ANGPTL4 to alter the cytoskeleton and degrade TJ proteins, leading to BBB breakdown. In addition, Egr-1 is a key regulatory molecule in the induction of neuroinflammation as well as the activation of microglia and astrocytes by meningitic *E. coli*

Meningeal pathogens have the ability to breach the BBB and compromise its integrity, thereby inciting a robust inflammatory response that culminates in neuronal damage. The precise mechanisms of BBB penetration vary among different meningeal pathogens [32]; however, disruption of BBB integrity is an essential step for most meningitic bacteria to gain entry into the CNS [33]. TJ proteins, the most crucial structural component of BBB, play a pivotal role in promoting cellular polarity and barrier integrity while ensuring CNS microenvironment stability [34]. Meningitic pathogens can degrade TJ proteins or rearrange junctional complexes to enhance BBB permeability. For example, *N. meningitidis* triggers a specific cleavage of Occludin by releasing host matrix metalloprotease 8, leading to detachment of hBMEC and promoting the opening of the paracellular pathway [35]. GBS-infected hBMEC upregulated the expression of SNAIL1 via the ERK1/2/MAPK signaling cascade and lipoteichoic acid, leading to degradation of ZO-1, Occludin, and Claudin-5 and disruption of BBB integrity [36]. The surface structure of *S. suis*, comprising muramidase-released protein, factor H-binding protein, and *S. suis* protein endopeptidase O, is implicated in impeding BBB function by reducing TJ proteins [37]. α-hemolysin in meningitic *E. coli* attenuates the TGFβ1-TGFBRII-Smad2/3-Gli1/2 axis, leading to reduced expression of ZO-1 in Hbmec [38]. Furthermore, OmpA-mediated adhesion of meningitic *E. coli* to hBMEC induces activation of PKCα signaling, resulting in dissociation of β-catenin from cadherins and ultimately leading to breakdown of the BBB [39]. In this study, we demonstrate that the disruption of the BBB induced by meningitic *E. coli* is facilitated by the host transcription factor Egr-1, and we identify Egr-1 as a potential target for intervention in cases of *E. coli* meningitis (Fig. 9).

To explore the occupancy of Egr-1 at newly opened chromatin sites induced by bacteria, we analyzed Egr-1 ChIP-seq datasets obtained from hBMEC. Further bioinformatics analysis revealed the involvement of Egr-1 in cytoskeletal fiber alterations in hBMEC following infection. Actin is typically arranged at the periphery of endothelial cells, but under pathogenic stimuli, it reorganizes into stress fibers within the cytoplasm, leading to increased BBB permeability [28, 40]. Canonical Rho-GTPases, including Rac1, Cdc42, and RhoA, are a group of small proteins that regulate various intracellular processes ranging from cytoskeletal dynamics to cell-substrate and cell-cell adhesion [41]. Several meningeal pathogens utilize Rho GTPases to facilitate their infiltration across the BBB. *N. meningitidis* type IV pili facilitates the recruitment of the PAR3/PAR6/PKC polarity complex and ezrin, clustering of transmembrane proteins, and activation of the GTPase Cdc42, leading to increased barrier permeability and paracellular BBB crossing [42]. Further, the activation of RhoA and Rac1 in response to type III GBS is implicated in the invasion of hBMEC by GBS [43]. Notably, the proteins OmpA and IbeA of meningitic *E. coli* are implicated in Fak- and Stat3-mediated activation of Rac1, while FimH and CNF1 participate in RhoA activation [3]. In this study, we observed that the absence of Egr-1 in hBMEC cells provided complete protection against meningitic *E. coli*-induced cytoskeletal rearrangement, as evidenced by a comparison of F-actin fiber structures between infected WT and Egr-1^−/−^ cells. Importantly, we demonstrated that the activation of RhoA, Rac1, and Cdc42 in infected WT cells was largely abrogated in Egr-1- deficient cells.

Cytoskeletal rearrangements and neovascularization are closely associated with vascular permeability [44, 45]. Among the mRNAs involved in regulating the actin cytoskeleton, PDGFB exhibited the most significant fold change. PDGF-BB regulates BBB homeostasis and is essential for maintaining CNS stability [46]. However, it also acts as a vascular permeability factor that can induce endothelial barrier dysfunction in an ischemic stroke model, despite its neuroprotective effects [47]. Cocaine-induced PDGF-BB expression in hBMEC disrupts the BBB by downregulating ZO-1 expression [48]. Recently, we have discovered that meningitic *E. coli* induces a time-dependent increase in PDGF-BB levels in hBMEC, resulting in the disorganization of TJ proteins [30]. Conversely, ANGPTL4 and VEGFA exhibited the most significant fold change among the mRNAs associated with angiogenesis regulation. VEGFA presents an attractive target for modulating brain function at the neurovascular interface. Pathologically elevated levels of VEGFA lead to increased vessel permeability and leakage, thereby compromising the integrity of the BBB [49]. In the context of CNS inflammation, VEGFA and TYMP collaborate to downregulate Claudin-5 and Occludin expression [50]. Luminal VEGFA signaling induces upregulation of endothelial nitric oxide synthase isoform, which enhances vascular permeability and facilitates the rearrangement of endothelial cells to support sprouting angiogenesis [51]. Similarly, meningitic *E. coli* infection significantly upregulates VEGFA expression, which in turn negatively regulates TJ protein expression to increase BBB permeability via the TLR2-MAPK-ERK1/2 pathway [21]. The ANGPTL4 C-terminal domain appears to have an important role in angiogenesis and vascular hyperpermeability [52]. The disruption of endothelial continuity is initiated by a tumor-derived cANGPTL4 through direct interaction with integrin α5β1, VE-cadherin, and Claudin-5 in a temporally sequential manner [53]. In diabetic retinopathy, hypoxia-inducible factor-1 upregulates ANGPTL4 expression in Müller cells of the hypoxic retina, thereby promoting vascular permeability [54]. In this study, we propose that Egr-1 binds with the PDGFB, VEGFA, and ANGPTL4 promoters, thereby increasing the secretion of PDGF-BB, VEGF-A, and ANGPTL4. This increase in secretion may modulate the expression of TJ proteins and alter the molecular composition within TJ complexes of hBMEC during infection.

Additionally, Egr-1 serves as a pivotal regulator that plays a crucial role in initiating an inflammatory response [55, 56]. Egr-1 activation may modulate a diverse range of inflammation-related genes such as TNF-α, IL-6, IL-17 A, ICAM-1, CCL2, CD44, and TGF-β [57, 58]. Moreover, Egr-1 serves as an upstream regulator for the pivotal inflammatory pathway NF-κB. Egr-1 increases RXRa acetylation by modulating p300, thereby exacerbating cerebral injury in a rat model of intracerebral hemorrhage and dysfunction in BMECs via the STAT3/NF-κB pathway [59]. Age-related upregulation of Egr-1 promotes apoptosis of granulosa cells via the NF-κB pathway during follicular atresia in ovarian aging [60]. Insights into the role of Egr-1 in modulating inflammation have been gleaned from studies utilizing Egr-1-deficient mice. *Egr-1*^*−/−*^ mice exhibit protection against kidney disease through attenuation of renal proximal tubule injury and suppression of NF-κB activity [61]. In a study on acute pancreatitis, deficiency of Egr-1 was found to mitigate cerulein-induced inflammation and protect the pancreas by reducing cytotoxic and proinflammatory cytokines [62]. Similarly, studies conducted on *Egr-1*^*−/−*^ mice have revealed that the inappropriate induction of Egr-1 is a contributing factor to inflammation and brain damage following a stroke [63]. Our study findings provide support for the inhibitory effects of Egr-1 deficiency on cytokine and adhesion molecule production in the brains of mice challenged with meningitic *E. coli*, which also resulted in a reduction of infection-induced meningeal thickening. Notably, 60% of the *Egr-1*^*−/−*^ mice became asymptomatic and survived. Collectively, these findings provide support for the significant suppression of meningitis-induced production of proinflammatory cytokines and adhesion molecules, as well as improved survival resulting from Egr-1 deficiency.

## Conclusions

Our findings demonstrate that meningitic *E. coli* rapidly induces the expression of a crucial host transcription factor, Egr-1, which in turn binds to its regulatory elements to alter the cytoskeleton and degrade TJ proteins, ultimately leading to BBB breakdown. Furthermore, we have identified Egr-1 as a pivotal regulator of neuroinflammation elicited by meningitic *E. coli* infection. These results suggest that targeting Egr-1 may represent a promising therapeutic strategy for bacterial meningitis.

### Supplementary Information

**Additional file 1.**

## Data Availability

All data generated or analyzed during this study are included in this published article.

## Abbreviations

*E. coli*: *Escherichia coli*
BBB: Blood-brain barrier
Egr-1: Early growth response 1
CNS: Central nervous system
BMEC: Brain microvascular endothelial cells
TJ: Tight junction
ZO: Zona occludin
GBS: Group B *Streptococcus*
*N. meningitidis*: *Neisseria meningitidis*
Hib: *Haemophilus influenza* type B
*S. suis*: *Streptococcus suis*
VEGFA: Vascular endothelial growth factor A
PDGFB: Platelet derived growth factor subunit B
ANGPTL4: Angiopoietin like 4
hBMEC: Human BMEC
RIPA: Radio-immunoprecipitation assay
PBS: Phosphate buffer saline
WT: Wild-type
CFUs: Colony-forming units
PAK: p21-activated kinase 1
ChIP-seq: Chromatin immunoprecipitation sequencing
PCR: Polymerase chain reaction
GO: Gene ontology
NCBI: National center for biotechnology information
KEGG: Kyoto encyclopedia of genes and genomes
CRISPR: Clustered regularly interspaced short palindromic repeat
Cas9: CRISPR-associated protein 9
ELISA: Enzyme-linked immunosorbent assay
IL-1β: Interleukin-1β
TNF-α: Tumor necrosis factorα
CCL-2: Chemokine (C-C motif) ligand 2
CXCL-2: Chemokine (C-X-C motif) ligand 2
HOMER: Hypergeometric optimization of motif enrichment
ICAM-1: Intercellular adhesion molecule 1
VCAM-1: Vascular cell adhesion molecule 1
