# Supplementary material for: Egr-1 is a key regulator of the blood-brain barrier damage induced by meningitic Escherichia coli
Source: Cell Commun Signal. 2024 Jan 17;22:44. doi: 10.1186/s12964-024-01488-y (PMC10795328; doi:10.1186/s12964-024-01488-y)
Supplement: Supplementary file 1 — Additional file 1. [file 12964_2024_1488_MOESM1_ESM.docx]

**Supplementary information**

**Supplementary methods**

**Cerebrospinal fluid bacterial** **titer and leukocyte count assays**

The specific pathogen-free C57BL/6 mice were obtained from Laboratory Animal Services Center at Huazhong Agricultural University. Mice at 25 days of age were injected via tail vein with *E. coli* strain PCN033 at 1×10^7^ colony-forming units (CFUs) diluted in phosphate-buffered saline. The mice were anesthetized upon manifestation of severe neurological symptoms. Subsequently, the muscles connected to the foramen magnum were carefully dissected to fully expose the white dura mater. Finally, a micropipette was gently inserted through the dura mater into the cisterna magna in order to collect cerebrospinal fluid. The cerebrospinal fluid bacteria were quantified by appropriate dilutions and plating techniques. The number of leukocytes in the cerebrospinal fluid was determined using a cell counting chamber.

**Supplementary Figure**

**
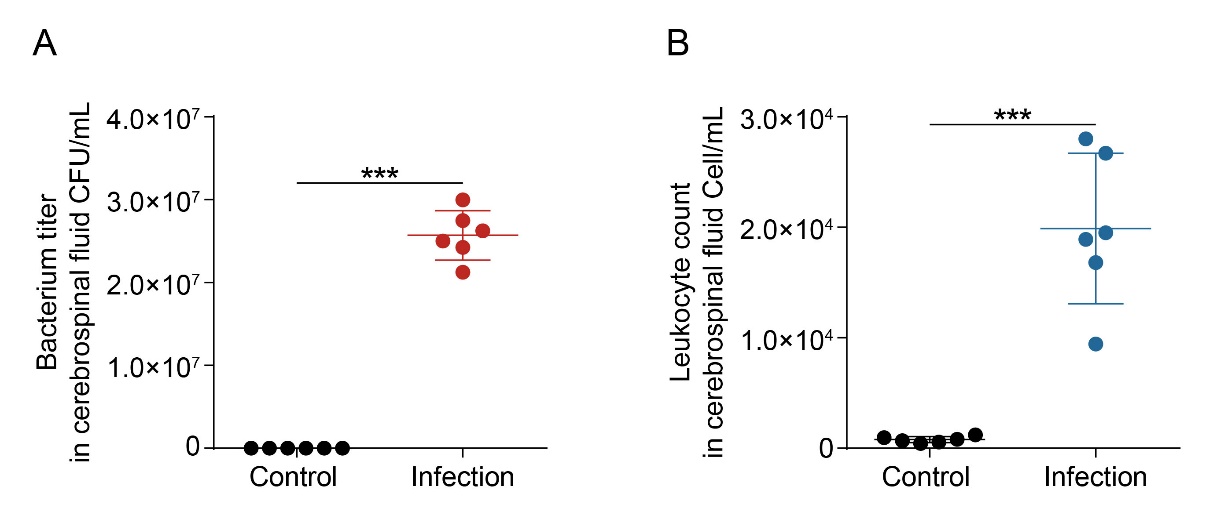
**

**Fig. S1 Bacterial titers and leukocyte counts in cerebrospinal fluid.** Mice were intravenously infected with meningitic *E. coli* (n = 6) at 1 × 10^7^ CFUs, and when they exhibited severe neurological symptoms, the bacterial loads (A) and leukocyte counts (B) in the cerebrospinal fluid were determined. Data are expressed as mean ± SD.

**Supplementary Tables**

**Table S1. Primers used in this study**

| Primers | Nucleotide sequence (5’-3’) |
| --- | --- |
| Human Egr-1 Real-time PCR | GGACAAGAAAGCAGACAA |
|  | GGATGGATAAGAGGTAGTAAC |
| Human VEGFA Real-time PCR | TGCCTTGCTGCTCTACCT |
|  | GACATCCATGAACTTCACCACTT |
| Human PDGFB Real-time PCR | GCTCTTCCTGTCTCTCTG |
|  | GGTCACTCAGCATCTCATA |
| Human ANGPTL4 Real-time PCR | GGCTCAGTGGACTTCAACCG |
|  | CCGTGATGCTATGCACCTTCT |
| Human ZO-1 Real-time PCR | TGTGGAAGAGGATGAAGATGAAGA |
|  | GGTGGAAGGATGCTGTTGTC |
| Human Occludin Real-time PCR | TTAACTTCGCCTGTGGAT |
|  | TGTGTAGTCTGTCTCATAGTG |
| Human Claudin-5 Real-time PCR | CGCCTTCCTGGACCACAACAT |
|  | CCAGCACCGAGTCGTACACTT |
| Human β-actin Real-time PCR | CATGTACGTTGCTATCCAGGC |
|  | CTCCTTAATGTCACGCACGAT |
| Mouse Egr-1 Real-time PCR | TCGGCTCCTTTCCTCACTCA |
|  | CTCATAGGGTTGTTCGCTCGG |
| Mouse ZO-1 Real-time PCR | GTCCCTCCTCTGATACCTTCCTC |
|  | CTGGCAGTGTCATTCACATCTTTC |
| Mouse Occludin Real-time PCR | ATGGAGGCTATGGCTATGG |
|  | GGAAGCGATGAAGCAGAAG |
| Mouse Claudin-5 Real-time PCR | AAAGGGCTCATTGCGGTGGTTGT |
|  | CTCGCTGTTGGAGTTCAGAAGTGGA |
| Mouse β-actin Real-time PCR | CACTGCCGCATCCTCTTCCTCCC |
|  | CAATAGTGATGACCTGGCCGT |
| Human VEGFA ChIP-PCR | TCAGGCTGTGAACCTTGGTGGG |
|  | CTCCCCGCTACCAGCCGACTTTTAA |
| Human PDGFB ChIP-PCR | GCAGAGGCCTGAGCGCCTGATC |
|  | GCAGCGATTCATGCCGACTCCG |
| Human ANGPTL4 ChIP-PCR | GGAAGTCTTGACCTCAAGCGTGCGC |
|  | CAGGCCTTCCTCTACGAACCGTGGG |
| Human Egr-1 sgRNAs | ACCTGCGGTCAGGTGCTCGT |
|  | TGCTCGCCCGTGTCCGCCTG |

**Table S2. Overlapping of differentially expressed genes and Egr-1 unique binding genes after meningitic *E. coli* infection in hBMEC**

| **Symbol** | **Ensembl** | **Base Mean** | **log2 Fold Change** | **P value** | **P adj** |
| --- | --- | --- | --- | --- | --- |
| ACSS2 | ENSG00000131069 | 6847.391318 | 1.293219168 | 2.34E-73 | 5.03E-71 |
| ANGPTL4 | ENSG00000167772 | 872.2278916 | 3.997383799 | 2.10E-223 | 2.65E-220 |
| ANKRD37 | ENSG00000186352 | 875.288933 | 5.468393354 | 2.16E-266 | 4.36E-263 |
| ARID3B | ENSG00000179361 | 2306.539656 | 1.346662806 | 2.02E-61 | 3.23E-59 |
| BBC3 | ENSG00000105327 | 956.0978819 | 1.832860504 | 1.40E-68 | 2.76E-66 |
| BHLHE40 | ENSG00000134107 | 13572.46414 | 2.992673271 | 1.41E-284 | 3.16E-281 |
| BHLHE41 | ENSG00000123095 | 15.30214649 | 1.230366267 | 0.010528268 | 0.028078167 |
| C4orf51 | ENSG00000237136 | 25.20139328 | -2.332010434 | 6.16E-08 | 5.22E-07 |
| CAMKK1 | ENSG00000004660 | 486.7142011 | 1.075795555 | 1.46E-20 | 4.99E-19 |
| CARD10 | ENSG00000100065 | 4591.961799 | 1.207113175 | 9.05E-59 | 1.39E-56 |
| CD83 | ENSG00000112149 | 600.0411201 | 1.733704367 | 3.71E-62 | 6.08E-60 |
| CRAMP1 | ENSG00000007545 | 360.4819454 | 1.000058258 | 3.88E-15 | 8.24E-14 |
| CRIM1 | ENSG00000150938 | 20793.09345 | 1.315531957 | 2.14E-56 | 3.06E-54 |
| CYP51A1 | ENSG00000001630 | 88.51564743 | -1.118124617 | 4.46E-07 | 3.26E-06 |
| DBP | ENSG00000105516 | 541.1873242 | 1.28402617 | 3.85E-29 | 2.08E-27 |
| DHRS13 | ENSG00000167536 | 117.8752863 | 1.609476123 | 1.32E-17 | 3.61E-16 |
| DNAJC28 | ENSG00000177692 | 58.66046915 | -1.196793037 | 6.39E-06 | 3.77E-05 |
| DUSP5 | ENSG00000138166 | 3470.523603 | 2.410031288 | 4.92E-209 | 5.22E-206 |
| EFNA3 | ENSG00000143590 | 114.8346302 | 1.532110384 | 9.36E-15 | 1.91E-13 |
| EGR1 | ENSG00000120738 | 9595.960875 | 5.220164105 | 0 | 0 |
| EGR3 | ENSG00000179388 | 18.06366866 | 6.775256568 | 4.25E-08 | 3.71E-07 |
| EHD1 | ENSG00000110047 | 3065.126961 | 1.121581754 | 9.95E-47 | 1.14E-44 |
| EPAS1 | ENSG00000116016 | 1206.826385 | 1.980026305 | 2.12E-111 | 8.54E-109 |
| EPC2 | ENSG00000135999 | 3910.424265 | -1.339160677 | 2.34E-67 | 4.45E-65 |
| EPHA2 | ENSG00000142627 | 6483.370896 | 1.730366733 | 7.01E-118 | 2.95E-115 |
| F3 | ENSG00000117525 | 1507.6202 | 1.089737684 | 8.17E-42 | 7.45E-40 |
| FAIM | ENSG00000158234 | 526.1139485 | -1.022649165 | 3.08E-23 | 1.24E-21 |
| FAM210A | ENSG00000177150 | 3381.651155 | 1.725293686 | 1.24E-109 | 4.82E-107 |
| FOSL2 | ENSG00000075426 | 3701.450824 | 1.926411855 | 6.35E-152 | 3.46E-149 |
| FSTL3 | ENSG00000070404 | 799.1913416 | 1.006545037 | 3.60E-19 | 1.11E-17 |
| GADD45B | ENSG00000099860 | 1319.866243 | 1.72841981 | 4.26E-55 | 5.92E-53 |
| GGT7 | ENSG00000131067 | 3049.060379 | 1.003594526 | 8.01E-46 | 8.83E-44 |
| GIPR | ENSG00000010310 | 397.3919399 | 1.56133561 | 1.13E-40 | 1.01E-38 |
| GPI | ENSG00000105220 | 9321.418031 | 1.281368281 | 5.12E-65 | 9.21E-63 |
| GPRC5A | ENSG00000013588 | 4924.220127 | 1.629023121 | 1.70E-134 | 7.99E-132 |
| GUK1 | ENSG00000143774 | 2790.641762 | 1.080100464 | 1.83E-34 | 1.26E-32 |
| HES7 | ENSG00000179111 | 683.5219438 | 1.749109213 | 6.69E-43 | 6.33E-41 |
| HIC1 | ENSG00000177374 | 92.25830314 | 1.056794203 | 1.42E-06 | 9.53E-06 |
| IFNGR2 | ENSG00000159128 | 1793.199518 | 1.05856938 | 1.31E-44 | 1.36E-42 |
| IL1R1 | ENSG00000115594 | 63.88663864 | -1.065942649 | 1.24E-05 | 6.90E-05 |
| ING3 | ENSG00000071243 | 861.0494867 | -1.533395107 | 7.22E-64 | 1.24E-61 |
| KATNAL2 | ENSG00000167216 | 58.01789116 | -2.807243767 | 2.11E-19 | 6.57E-18 |
| KDM3A | ENSG00000115548 | 6473.005959 | 1.840057976 | 4.93E-135 | 2.43E-132 |
| KDM6B | ENSG00000132510 | 2563.628706 | 1.698648604 | 9.65E-91 | 2.90E-88 |
| KIAA0040 | ENSG00000235750 | 69.88217149 | -1.128455187 | 1.93E-06 | 1.26E-05 |
| KISS1R | ENSG00000116014 | 48.35019393 | 1.317936888 | 4.78E-06 | 2.91E-05 |
| KLF10 | ENSG00000155090 | 3856.192423 | 1.431596823 | 1.16E-75 | 2.52E-73 |
| KLF6 | ENSG00000067082 | 19138.36656 | 1.424210494 | 7.35E-79 | 1.81E-76 |
| KLHL32 | ENSG00000186231 | 59.2991531 | -2.72705981 | 1.11E-18 | 3.29E-17 |
| KREMEN2 | ENSG00000131650 | 122.6201102 | 1.194621775 | 2.16E-10 | 2.62E-09 |
| LHX4 | ENSG00000121454 | 195.7821152 | -1.050310571 | 4.66E-11 | 6.17E-10 |
| LMNTD2 | ENSG00000185522 | 227.5724533 | 1.005351981 | 1.30E-11 | 1.84E-10 |
| LOXL2 | ENSG00000134013 | 3964.300377 | 1.014601481 | 4.02E-49 | 4.94E-47 |
| MAFF | ENSG00000185022 | 609.8523901 | 2.293288147 | 2.54E-78 | 5.96E-76 |
| MICALL2 | ENSG00000164877 | 398.5466111 | 1.208827329 | 4.15E-22 | 1.57E-20 |
| MYC | ENSG00000136997 | 3448.369293 | 2.049094459 | 2.44E-117 | 1.00E-114 |
| NAB2 | ENSG00000166886 | 1909.207878 | 2.149935318 | 8.36E-98 | 2.72E-95 |
| NEDD4 | ENSG00000069869 | 14012.87257 | 1.171532533 | 1.78E-41 | 1.60E-39 |
| NFKB1 | ENSG00000109320 | 4374.77015 | 1.130989592 | 2.32E-45 | 2.49E-43 |
| NFKBID | ENSG00000167604 | 906.4290158 | 1.875102499 | 2.73E-78 | 6.33E-76 |
| NFKBIE | ENSG00000146232 | 442.3087992 | 1.703210962 | 1.80E-45 | 1.94E-43 |
| NINJ1 | ENSG00000131669 | 402.7931755 | 1.150098212 | 1.55E-22 | 6.01E-21 |
| NIPAL2 | ENSG00000104361 | 18.50264596 | -1.78036573 | 0.000185825 | 0.000801068 |
| NOCT | ENSG00000151014 | 845.1307746 | 2.883345056 | 9.67E-155 | 5.57E-152 |
| NR4A1 | ENSG00000123358 | 1578.69884 | 2.861948512 | 5.88E-193 | 5.15E-190 |
| NR4A2 | ENSG00000153234 | 802.6474554 | 1.389566617 | 1.70E-45 | 1.84E-43 |
| NUP58 | ENSG00000139496 | 3468.418283 | 1.019753107 | 1.36E-40 | 1.21E-38 |
| ODAD1 | ENSG00000105479 | 128.2667397 | 1.155974621 | 3.27E-10 | 3.87E-09 |
| ONECUT1 | ENSG00000169856 | 48.45917017 | -1.250159072 | 1.34E-05 | 7.44E-05 |
| PDGFB | ENSG00000100311 | 4424.162272 | 1.360960999 | 1.58E-67 | 3.03E-65 |
| PER1 | ENSG00000179094 | 1391.878208 | 1.819338638 | 6.65E-97 | 2.13E-94 |
| PFKP | ENSG00000067057 | 13158.78195 | 1.329592351 | 1.11E-89 | 3.16E-87 |
| PNCK | ENSG00000130822 | 22.71678681 | 1.304079582 | 0.001073044 | 0.00381027 |
| PPARGC1B | ENSG00000155846 | 1832.041894 | 1.022353063 | 5.42E-38 | 4.42E-36 |
| PPP1R13L | ENSG00000104881 | 4396.887128 | 1.283522939 | 6.30E-77 | 1.44E-74 |
| PPP1R18 | ENSG00000146112 | 4136.048253 | 1.121920365 | 2.53E-30 | 1.44E-28 |
| PRR5-ARHGAP8 | ENSG00000248405 | 32.70683243 | 1.650612014 | 1.47E-06 | 9.82E-06 |
| PTP4A2 | ENSG00000184007 | 1017.83183 | 1.639135863 | 2.69E-62 | 4.44E-60 |
| RAB20 | ENSG00000139832 | 341.2784618 | 1.69805923 | 4.38E-44 | 4.40E-42 |
| RARA | ENSG00000131759 | 2434.787978 | 2.348397816 | 8.25E-174 | 6.16E-171 |
| RLF | ENSG00000117000 | 4760.412426 | 1.340745101 | 9.39E-58 | 1.42E-55 |
| RNF24 | ENSG00000101236 | 2189.557344 | 1.072943029 | 1.34E-31 | 8.12E-30 |
| SDC4 | ENSG00000124145 | 3337.865782 | 1.726091564 | 7.68E-105 | 2.72E-102 |
| SENP8 | ENSG00000166192 | 172.6393592 | -1.051810554 | 6.21E-11 | 8.10E-10 |
| SERPINI1 | ENSG00000163536 | 139.4072566 | -1.26413076 | 1.75E-13 | 3.08E-12 |
| SH3GL3 | ENSG00000140600 | 160.2331726 | 1.143110854 | 5.87E-12 | 8.60E-11 |
| SLC2A1 | ENSG00000117394 | 2304.672133 | 2.425013702 | 2.92E-202 | 2.80E-199 |
| SLC7A5 | ENSG00000103257 | 13630.52959 | 1.126176725 | 1.14E-56 | 1.65E-54 |
| SLCO4A1 | ENSG00000101187 | 32588.65446 | 1.409459384 | 3.18E-102 | 1.07E-99 |
| SMAD7 | ENSG00000101665 | 1859.61447 | 1.380212975 | 6.97E-64 | 1.21E-61 |
| SPAG4 | ENSG00000061656 | 598.821649 | 1.298021237 | 1.84E-36 | 1.41E-34 |
| SPESP1 | ENSG00000258484 | 121.0654196 | -1.505690937 | 5.25E-15 | 1.10E-13 |
| SSBP2 | ENSG00000145687 | 222.8408302 | 1.04875487 | 2.25E-11 | 3.09E-10 |
| ST3GAL1 | ENSG00000008513 | 1968.576337 | 1.703167352 | 3.19E-103 | 1.11E-100 |
| TACR2 | ENSG00000075073 | 28.24100248 | -1.038499644 | 0.003736476 | 0.011459513 |
| TBC1D10A | ENSG00000099992 | 668.6364308 | 1.259790446 | 7.34E-37 | 5.67E-35 |
| TBC1D2 | ENSG00000095383 | 2445.196399 | 1.000680147 | 2.90E-44 | 2.95E-42 |
| TIPARP | ENSG00000163659 | 10730.22184 | 1.226674133 | 1.35E-54 | 1.85E-52 |
| TMEM170B | ENSG00000205269 | 423.3061009 | -1.021027142 | 4.66E-18 | 1.32E-16 |
| TNFRSF12A | ENSG00000006327 | 1010.228035 | 1.114405294 | 1.70E-18 | 4.94E-17 |
| TPTEP2-CSNK1E | ENSG00000283900 | 91.3389396 | 1.142799852 | 3.25E-08 | 2.88E-07 |
| TSC22D3 | ENSG00000157514 | 326.662764 | 1.167134385 | 5.33E-20 | 1.75E-18 |
| URGCP-MRPS24 | ENSG00000270617 | 19.47502903 | -2.039073252 | 2.54E-05 | 0.000132737 |
| USP43 | ENSG00000154914 | 1175.526671 | 1.727774553 | 2.33E-90 | 6.90E-88 |
| VEGFA | ENSG00000112715 | 2829.469626 | 3.191429658 | 0 | 0 |
| WWC1 | ENSG00000113645 | 4805.131564 | 1.106049885 | 3.38E-50 | 4.29E-48 |
| YPEL2 | ENSG00000175155 | 1837.13479 | 1.214167124 | 1.61E-49 | 2.01E-47 |
| ZNF160 | ENSG00000170949 | 5048.906094 | 1.33843763 | 1.25E-85 | 3.40E-83 |
| ZNF775 | ENSG00000196456 | 48.35279368 | -1.193719055 | 7.55E-05 | 0.00035502 |
| ZNF816-ZNF321P | ENSG00000221874 | 204.6105919 | 1.158146067 | 5.97E-15 | 1.24E-13 |
| ZNRF1 | ENSG00000186187 | 3181.960093 | 1.283523701 | 2.91E-48 | 3.50E-46 |
